# Lower Vitamin D Levels, but Not VDR Polymorphisms, Influence Type 2 Diabetes Mellitus in Brazilian Population Independently of Obesity †

**DOI:** 10.3390/medicina55050188

**Published:** 2019-05-22

**Authors:** Kathryna Fontana Rodrigues, Nathalia Teixeira Pietrani, Adriana Aparecida Bosco, Maira Cândida Rodrigues de Sousa, Ieda de Fátima Oliveira Silva, Josianne Nicácio Silveira, Karina Braga Gomes

**Affiliations:** 1 Instituto de Ciências Biológicas, Universidade Federal de Minas Gerais, Belo Horizonte, MG 31270-901, Brazil; katyfontanar@gmail.com (K.F.R.); ntpietrani@hotmail.com (N.T.P.); 2 Instituto de Ensino e Pesquisa, Santa Casa de Belo Horizonte, Belo Horizonte, MG 30150-240, Brazil; a.bosco@terra.com.br; 3 Faculdade de Farmácia, Universidade Federal de Minas Gerais, Belo Horizonte, MG 31270-901, Brazil; maira.crsousa@gmail.com (M.C.R.d.S.); iedafos@farmacia.ufmg.br (I.d.F.O.S.); nicacio@farmacia.ufmg.br (J.N.S.)

**Keywords:** vitamin D, obesity, type 2 diabetes mellitus, vitamin D receptor, polymorphisms

## Abstract

*Background and Objectives*: Vitamin D levels have been associated with a diversity of diseases, including obesity. Vitamin D presents a pleiotropic action, and can regulate insulin secretion and inflammatory responses. Vitamin D receptor (*VDR*) gene polymorphisms are involved in the gene expression regulation and have been associated with type 2 diabetes mellitus (T2DM). This study aimed to evaluate the association between the polymorphisms ApaI (rs7975232), BsmI (rs1544410), FokI (rs10735810), and TaqI (rs731236) in the *VDR* gene in people diagnosed with T2DM, and plasma 25-hydroxivitamin D levels [25(OH)D]. *Materials and Methods*: A total of 101 T2DM patients and 62 gender, age, and body mass index (BMI) matched non-diabetic controls were included in this study. Molecular analyzes were performed by polymerase chain reaction–restriction fragment length polymorphism (PCR-RFLP). The plasma 25(OH)D levels were measured by high performance liquid chromatography. *Results*: The plasma 25(OH)D levels were lower in T2DM patients (17.2 (16.6) ng/mL) when compared with the control subjects (30.8 (16.2) ng/mL, *p* < 0.0001), independently of obesity status. We found no difference between genotypic and allelic frequencies of the *VDR* polymorphisms when comparing the T2DM group and control group (*p* > 0.05 for all), and did not show any association with plasma 25(OH)D levels. *Conclusions*: These results suggest that T2DM is associated with lower plasma 25(OH)D levels, which are not related to BMI and *VDR* gene polymorphisms.

## 1. Introduction

Type 2 diabetes mellitus (T2DM) is the most common type of diabetes, accounting for around 90% of all cases of diabetes. According to the International Diabetes Federation, there were 424.9 million people in the world with diabetes in 2017, and this number is expected to reach 628.6 million by 2045, with approximately 50% of cases being undiagnosed. Currently, Brazil ranks 4th in countries with the highest number of adults with diabetes mellitus [1]. T2DM is a chronic disease and its pathogenesis has been associated with a subclinical inflammation and activation of the immune system [2].

Vitamin D (VitD) seems to play an important role in the regulation of insulin secretion, since it controls the calcium concentration and its flux through the beta cells [3]. In addition, 1,25(OH)_2_D stimulates transcription of the insulin receptor gene, increasing the number of receptors available [4], and activates the transcriptional factor PPAR-δ that regulates free fatty acids metabolism in muscle and adipose tissues, increasing insulin sensitivity [5]. VitD influences the anti-inflammatory response in T2DM because it inhibits the NF-κB pathway, and stimulates regulatory T cell development and Th2 phenotype induction [6].

About 3% of the human genome can be regulated by VitD [7,8]. VitD is synthesized by the skin through exposure to sunlight, and a small portion comes from dietary sources. VitD exerts its biological actions by the interaction to its receptor (VDR), which is a member of the steroid receptor superfamily with a DNA binding domain. The *VDR* gene is located on chromosome 12q13.1 and presents 11 exons [7]. Single nucleotide polymorphisms (SNPs) in the *VDR* gene, such as ApaI (C/A, rs795232, intron 8), BsmI (G/A, rs1544410, íntron 8), FokI (C/T, rs10735810, exon 2), and TaqI (T/C, rs731236, exon 9), may interfere in the VitD –VDR complex formation, with consequent reduction of VitD absorption, even in the presence of normal serum levels [9,10,11,12].

In spite of the existence of some studies examining the association between VitD levels, *VDR* polymorphisms, and T2DM in different populations, the results are not clear [13,14,15,16,17,18,19,20,21,22,23,24,25]. Therefore, in this study we aimed to evaluate the association between 25-hydroxy vitamin D [25(OH)D] levels and polymorphisms in the *VDR* gene (ApaI, BsmI, FokI, and TaqI) in a group of T2DM Brazilian patients, according to their body mass index (BMI).

## 2. Materials and Methods

### 2.1. Ethical Aspects

All participants signed an informed consent form, which was approved on 6 May 2011 by the Ethics Committees of the Federal University of Minas Gerais (Minas Gerais, Brazil, ETIC 0062.0.203.000-11) and Santa Casa Hospital (Minas Gerais, Brazil, 059/2011) in accordance with the Helsinki Declaration.

### 2.2. Experimental Design

This case-control study included 101 patients with T2DM diagnosis, according to the American Diabetes Association (ADA) [26], and 62 gender, age and BMI matched non-diabetic individuals, aged from 32 to 70 years. According to a sample calculation based on the mean values of VitD levels from a sample of the groups (power = 0.95, significance level = 0.05), a 1:1 case/control proportion was adopted. More T2DM patients were included in the sample to account for other variables, such as co-morbidities, use of medications and clinical outcomes. The patients were recruited at the Santa Casa Hospital (Minas Gerais, Brazil) from June 2012 to September 2013. Control group subjects were recruited from the local community during the same period. The exclusion criteria were: current or recent infections and/or inflammatory processes, recent history of cardiovascular disease (e.g., heart attack, stroke, thrombosis in the last five years), age over 70 years, autoimmune diseases, pregnancy, and cancer. Interviews and medical records were utilized to obtain clinical and laboratorial data for all T2DM patients. Individuals who used supplements containing VitD six months before the sample collection were not included in this study.

### 2.3. Blood Sampling

Blood samples were collected in EDTA, heparin sodium, and anticoagulant-free tubes. The blood samples were then centrifuged at 1100× *g* for 20 min at 25 °C. The plasma or serum was stored at −80 °C until biochemical analysis. An aliquot of whole blood collected in EDTA was used for genomic DNA extraction and *VDR* polymorphisms genotyping.

### 2.4. Biochemical Measurements

Fasting glucose levels for T2DM group were obtained from updated medical records, considering the result presented on the day of the recruitment. For the control group, the glucose blood levels were measured by the enzyme-colorimetric method (Glucose, Gold Analisa and BTR 811 spectrophotometer, Biotron, Brazil).

The high-sensitivity C reactive protein (hs-CRP) levels were measured in serum samples using the immunoturbidimetric method (hs-CRP, VITROS Chemistry Products and System Vitros Chemistry 5.1 FS, Ortho Clinical Diagnostics, Raritan, NJ, USA).

The reversed-phase high performance liquid chromatography (HPLC) with UV detection (Thermo Finnigan Surveyor, Waltham, MA, USA) was used to measure the 25(OH)D levels in the EDTA plasma samples, according to Hymoller and Jensen [27] modified. The 25(OH)D_2_ (≥98% pure) and 25(OH)D_3_ (≥99% pure) were used as standards and 1,α-hydroxyvitamin D_3_ (1αOHD_3_—≥97% pure) as an internal standard (all standards from Sigma Aldrich^®^, Burlington, MA, USA). The detection-limits obtained in the study were: 9.6 ng/mL for 25(OH)D_3_ and 10.6 ng/mL for 25(OH)D_2_. The linearity was 200 ng/mL for both metabolites. Individuals were classified as deficient when the plasma 25(OH)D levels were from <20 ng/mL, insufficient from 20 to 30 ng/mL, and sufficient from >30 ng/mL [28].

### 2.5. VDR Polymorphisms Genotyping

Genomic DNA samples were obtained from whole blood using the Biopur Mini Spin Kit (Biometrix Biotecnologia^®^, Curitiba, Paraná, Brazil). The polymorphisms on the *VDR* gene (ApaI, BsmI, FokI, and TaqI) were genotyped by polymerase chain reaction–restriction fragment length polymorphism (PCR-RFLP) using the restriction enzymes (ApaI, BsmI, FokI, and TaqI respectively) according to Ranjzad et al. [29]. This was followed by electrophoresis in a 6% polyacrylamide gel stained with silver nitrate.

### 2.6. Statistical Analysis

The data were tested for normality using the Shapiro–Wilk test. Parametric data were presented as a mean ± standard deviation (SD), non-parametric data as a median [interquartile range (IQR)], and categorical data as an absolute value (percentage of total).

Comparisons for the parametric variables were performed with a Student’s *t* test. For non-parametric variables we used the Mann–Whitney or the Kruskal–Wallis test, and the chi-square (x^2^) test for categorical variables. Correlations were assessed using Spearman’s correlation test.

The Hardy–Weinberg equilibrium (HWE) was evaluated using an exact test by GENEPOP on-line software (http://genepop.curtin.edu.au). The differences in genotypic and allelic frequencies between the groups were tested with the chi-square (x^2^) test.

Statistical analysis was performed with the Statistical Package of the Social Sciences (SPSS) version 17.0 (SPSS Inc., Chicago, IL, USA). For all analyses a *p*-value of <0.05 was considered statistically significant.

## 3. Results

Table 1 presents the clinical and laboratorial characterization of case and control groups, which were matched by gender, age, and obesity status (a BMI of < 30 Kg/m^2^ - non-obese, a BMI of ≥ 30 Kg/m^2^ - obese, and *p* > 0.05 for all). T2DM patients displayed higher waist circumference (*p* < 0.0001) and fasting glucose (*p* < 0.0001) when compared to the non-diabetic control group.

Plasma 25(OH)D levels were lower in T2DM patients (17.2 (16.6) ng/mL) than in the control group (30.8 (16.2) ng/mL, *p* < 0.0001) (data presented at: XXI Congress of the Brazilian Society of Diabetes [30]). Considering the plasma 25(OH)D levels categorization (deficient when levels were <20 ng/mL, insufficient from 20 to 30 ng/mL, and sufficient at >30 ng/mL), 59.7% of T2DM patients and 12.0% of control subjects presented with a plasma 25(OH)D level deficiency. Moreover, patients with a plasma deficiency (10.6 (8.0) ng/mL) and an insufficiency (23.5 (5.9) ng/mL) exhibited even lower plasma 25(OH)D levels than the control group with a deficiency (17.2 (5.0) ng/mL, *p* = 0.015) and an insufficiency (26.7 (3.1) ng/mL, *p* = 0.011). No differences were found when comparing the T2DM patients (49.6 (25.5) ng/mL) and the control group (40.6 (14.5) ng/mL) with sufficient plasma 25(OH)D levels (*p* = 0.138).

Analyses of plasma 25(OH)D levels were performed when considering the subjects obesity status. The T2DM patients with a BMI of < 30 Kg/m^2^ and BMI ≥ 30 Kg/m^2^ exhibited lower plasma 25(OH)D levels (17.1 (13.8) ng/mL and 19.1 (19.1) ng/mL, respectively) when compared with the control group with a BMI of < 30 Kg/m^2^ and a BMI of ≥ 30 Kg/m^2^ (29.7 (14.3) ng/mL and 35.0 (22.2) ng/mL, respectively). However, no significant differences were observed when comparing the plasma 25(OH)D levels between non-obese and obese subjects within the same group [non-obese T2DM = 17.1 (13.8) ng/mL × obese T2DM = 19.1 (19.1) ng/mL; and non-obese controls = 29.7 (14.3) ng/mL × obese controls = 35.0 (22.2) ng/mL] (Table 2).

The genotypic and allelic frequencies of the *VDR* gene polymorphisms are presented in Table 3, which are in Hardy–Weinberg equilibrium (*p* > 0.025) for either groups [30]. No polymorphism showed a significant association with T2DM (*p* > 0.05 for all). Plasma 25(OH)D levels also did not show association with the genotypes of the ApaI, BsmI, FokI, and TaqI polymorphisms, considering all the groups or only T2DM group (*p* > 0.05 for all - Table 4).

Finally, plasma 25(OH)D levels showed a significant and negative, but a weak correlation with fasting glucose (r = −0.343, *p* < 0.0001).

## 4. Discussion

This study evaluated the association between plasma 25(OH)D levels and *VDR* gene polymorphisms in T2DM patients. The data demonstrated that lower 25(OH)D levels are associated with T2DM, which are not influenced by obesity status and *VDR* gene polymorphisms (ApaI, BsmI, FokI, and TaqI). Besides, these polymorphisms are not associated with T2DM.

The diabetic group exhibited lower plasma 25(OH)D levels than the control group. In agreement, some cross-sectional case-control studies also have demonstrated the association of lower plasma 25(OH)D levels and T2DM [13,18,19,21]. We found a negative correlation between plasma 25(OH)D levels and fasting glucose levels. Similarly, Clemente−Postigo et al. [19] in a cohort study also found an inverse correlation between VitD levels, insulin resistance (measured by the HOMA-IR index) and glycaemia. A recent systematic review and meta-analysis concluded that hypovitaminosis D is associated with increased risk of hyperglycemia both in diabetic and non-diabetic subjects [31]. However, Gendy et al. [32] did not find significant association between VitD levels with glycemic control, fasting lipids, and BMI in patients with T2DM from Egypt, although VitD deficiency was more prevalent in patients with T2DM. Indeed, a randomized clinical trial about VitD supplementation showed no demonstrated significant effect on controlling fasting glucose level, improving insulin resistance, or preventing T2DM in non-diabetic individuals [33].

Obesity status did not influence plasma 25(OH)D levels in both groups. On the contrary, Rafiq and Jeppesen [34] showed evidence that VitD levels present an inverse relationship with BMI in diabetic and non-diabetic subjects. VitD and its metabolites are liposoluble, and thus circulating excess of plasma 25(OH)D is attracted to adipose tissue and converted into inactive products [35].

It is known that the active form of VitD, 1,25 dihydroxyvitamin D [1,25(OH)_2_D], exerts its biological effects mediated by the VDR and there is a relationship, not completely elucidated, between VitD concentration and *VDR* gene polymorphisms. Martineau et al. [36] demonstrated that individuals with the TT genotype for TaqI polymorphism in the *VDR* gene showed a different response to VitD supplementation when compared with carriers of other genotypes (TC and CC). However, in the present study, the polymorphisms ApaI, BsmI, FokI, and TaqI did not show an association with T2DM, and had no influence on VitD levels. Similarly, Wang et al. [14] and Li et al. [16] in a meta-analysis also found no association between these polymorphisms and T2DM. However, Safar et al. [24] in a study conducted in the Emirati population found association between FokI and BsmI SNPs with T2DM. Malik et al. [23] observed an association between TaqI (T allele) and BsmI (G allele) polymorphisms with T2DM in Northern Indians, and Dong et al. showed an association with FokI polymorphism in the Han population from the south of China [37]. A meta-analysis conducted by Wang et al. [38] showed that BsmI polymorphism may be a risk factor for susceptibility to type 1 diabetes mellitus (T1DM) in the East Asian population, and the FokI polymorphism is associated with an increased risk of T1DM in the West Asian population. Contrary, Angel et al. [39] did not support the hypothesis of a significant contribution of Bsml, Apal and Taql VDR polymorphisms in the etiology of T1DM of Chilean patients. Interestingly, the study of Sarma et al. [25] in T2DM patients from north eastern India showed that body weight and BMI were significantly associated with BsmI and TaqI polymorphisms. BsmI was associated with HbA1C, and the frequency of the heterozygous genotype of the BsmI polymorphism was significantly higher in T2DM patients than in controls. Contradictory results between the studies may be due to several factors, including genetic background and use of VitD supplementation. Therefore, larger scale studies are required to better elucidate the possible association between *VDR* SNPs and T2DM.

This study presented some limitations such as a small sample size, chances of selection, as well as recall and misinformation bias due to its retrospective nature. The polymorphisms selected in our study do not provide complete coverage of the SNPs present in the *VDR* gene, so we cannot rule out that other genetic variants of *VDR* may be associated with T2DM. Indeed, this is a cross-sectional study and prospective analysis should be conducted in order to evaluate if low plasma levels of 25(OH)D and T2DM may represent reverse causality, that is, 25(OH)D deficiency is a consequence and not a cause of T2DM.

Our study presented strict selection criteria for patients and controls and it showed, for the first time, the relationship between plasma 25(OH)D levels and VDR polymorphisms in the Brazilian T2DM population.

## 5. Conclusions

In conclusion, our results suggest that Brazilian T2DM patients presented lower plasma 25(OH)D levels, which were not affected by obesity and polymorphisms in the *VDR* gene. Therefore, our data will contribute to the knowledge about the laboratorial and molecular profile of VitD in Brazilian T2DM patients.

## Figures and Tables

**Table 1:** Clinical and laboratory characterization of type 2 diabetes mellitus and control groups.

Variables	T2DM	Control	*p*
Gender			
Male (n)	19 (18.8%)	12 (19.4%)	0.932
Female (n)	82 (81.2%)	50 (80.6%)	
Age (years)	56 (13)	53 (18)	0.350
Obesity status			
BMI < 30 Kg/m^2^	25.5 (5.2)	24.8 (4.5)	0.204
BMI ≥ 30 Kg/m^2^	38.5 (9.4)	39.3 (11.0)	0.843
Waist circumference (cm)	108.1 ± 16.9	96.9 ± 16.2	<0.0001 *
Fasting glucose (mg/dL)	127.0 (82.0)	85.3 (10.0)	<0.0001 *
hs-CRP (mg/L)	2.8 (4.4)	2.6 (2.5)	0.405

T2DM (Type 2 diabetes mellitus). BMI (Body mass index). hs-CRP (High sensitivity C reactive protein). Categorical variable (gender) was presented as absolute value (percentage of total) - Chi-square (x^2^) test. Parametric variable (waist circumference) was presented as mean ± SD - Student’s *t* test. Non-parametric variables (age, obesity status, fasting glucose and hs-CRP) were presented as median (IQR) - Mann-Whitney test. * *p* < 0.05 was considered statistically significant.

**Table 2:** 25(OH)D levels according obesity status in type 2 diabetes mellitus and control groups.

Obesity Status	25(OH)D (ng/mL)		*p*
	T2DM	Control	
BMI < 30 Kg/m^2^	17.1 (13.8)	29.7 (14.3)	<0.0001 *
BMI ≥ 30 Kg/m^2^	19.1 (19.1)	35.0 (22.2)	0.001 *
*p*′	0.488	0.244	-

BMI (Body mass index). T2DM (Type 2 diabetes mellitus); * *p* < 0.05 was considered statistically significant (comparison between T2DM and control groups) - Mann–Whitney test; *p*′ < 0.05 was considered statistically significant (comparison inside T2DM or control groups) - Mann–Whitney test.

**Table 3:** Genotypic and allelic frequencies between the T2DM and control groups for the *VDR* gene polymorphisms.

Polymorphisms		T2DM	Control	*p*	OR	CI 95%
**ApaI** **(rs7975232)**	**Genotype**					
	AA	60 (59.4%)	38 (61.3%)	0.474	0.395	0.080–1.959
	AC	33 (32.7%)	22 (35.5%)		0.375	0.073–1.935
	CC	8 (7.9%)	2 (3.2%)		Reference	
	**Allele**					
	A	153 (75.7%)	98 (79.0%)	0.493	0.828	0.466–1.467
	C	49 (24.5%)	26 (21.0%)			
**BsmI** **(rs1544410)**	**Genotype**					
	AA	16 (15.8%)	9 (14.5%)	0.843	0.988	0.370–2.639
	AG	49 (48.5%)	33 (53.2%)		0.825	0.409–1.665
	GG	36 (35.6%)	20 (32.3%)		Reference	
	**Allele**					
	A	81 (40.1%)	51 (41.1%)	0.854	0.958	0.593–1.550
	G	121 (59.9%)	73 (58.9%)			
**FokI** **(rs10735810)**	**Genotype**					
	CC	61 (60.4%)	31 (50.0%)	0.430	1.530	0.521–4.499
	CT	31 (30.7%)	24 (38.7%)		1.005	0.327–3.086
	TT	9 (8.9%)	7 (11.3%)		Reference	
	**Allele**					
	C	153 (75.7%)	86 (69.4%)	0.206	1.380	0.812–2.342
	T	49 (24.5%)	38 (30.6%)			
**TaqI** **(rs731236)**	**Genotype**					
	CC	10 (9.9%)	9 (14.5%)	0.404	0.530	0.187–1.500
	CT	47 (46.5%)	32 (51.6%)		0.701	0.353–1.393
	TT	44 (43.6%)	21 (33.9%)		Reference	
	**Allele**					
	C	67 (33.2%)	50 (40.3%)	0.191	0.735	0.450–1.199
	T	135 (66.8%)	74 (59.7%)			

T2DM (Type 2 diabetes mellitus). The variables were presented as an absolute value (percentage of total). *p* < 0.05 was considered statistically significant; Chi-square (x^2^) test.

**Table 4:** 25(OH)D levels (ng/mL) according to *VDR* gene genotypes.

Polymorphisms		All Individuals	*p*	T2DM	*p*
ApaI (rs7975232)	AA	24.7 (22.7)	0.656	16.9 (16.7)	0.726
	AC	25.2 (17.5)		17.8 (16.5)	
	CC	17.9 (29.9)		14.6 (12.4)	
BsmI (rs1544410)	AA	26.0 (37.6)	0.415	20.5 (27.7)	0.468
	AG	24.8 (18.5)		17.3 (16.7)	
	GG	21.2 (19.3)		13.2 (12.5)	
FokI (rs10735810)	CC	23.2 (20.2)	0.764	17.4 (14.1)	0.213
	CT	27.4 (24.5)		12.3 (17.4)	
	TT	22.9 (35.2)		19.1 (41.4)	
TaqI (rs731236)	CC	20.3 (30.3)	0.222	19.1 (22.9)	0.635
	CT	25.6 (23.3)		17.2 (16.0)	
	TT	21.4 (19.3)		14.6 (16.30	

T2DM (Type 2 diabetes mellitus); *p* < 0.05 was considered statistically significant; Kruskal-Wallis test.
